# The Impact of the Aussie Optimism Program on the Emotional Coping of 5- to 6-Year-Old Children

**DOI:** 10.3389/fpsyg.2021.570518

**Published:** 2021-08-11

**Authors:** Selina Oorloff, Rosanna Rooney, Natalie Baughman, Robert Kane, Maryanne McDevitt, Aidan Bryant

**Affiliations:** School of Population Health, Faculty of Health Sciences, Curtin University, Perth, WA, Australia

**Keywords:** emotion regulation, early intervention, early childhood, internalizing disorders, prevention programs, externalizing disorders, emotional coping

## Abstract

Research indicates that mental health disorders can occur in children as young as 4 years of age, prompting the need for prevention programs for young children. The ability to use healthy strategies to cope with emotions is a protective factor against mental health disorders that can be effectively taught to children from an early age. The current study used a pre-test post-test cluster randomized controlled trial to test the efficacy of the new Aussie Optimism: I Spy Feelings Program. The aim of the study was to investigate the effects of the program on children’s emotional coping. The program included content on emotion regulation strategies, focusing on the emotions of happiness, sadness, anger, fear, and worry. The participants were 73 children (intervention = 33; control = 40) from pre-primary classes. Four schools were cluster randomized to the intervention or control group, resulting in two schools in each condition. Parents completed measures of their children’s emotional coping with sadness, anger and worry. Children in the intervention group participated in ten sessions of the I Spy Feelings Program, spread over 5 weeks. The results indicated a significant, small to moderate intervention effect for coping with anger. Children in the control group decreased in their ability to cope with anger, while children in the intervention group remained stable. No intervention effects were found for coping with sadness or worry, with results for these emotions staying stable across time for both groups. This pilot study will inform the further development of the program. The effects of the program on coping with anger provide support for the use of emotion regulation strategies in intervention programs to maintain healthy emotional coping, which is a protective factor against internalizing and externalizing disorders in childhood.

## Introduction

Mental illness is a significant contributor to the global burden of disease for children and young people (World Health Organization., 2019). Increasing rates of mental disorders have become a critical issue in recent years, especially with many disorders emerging early on in life (Olfson et al., 2014; Erskine et al., 2015). As reported in the Telethon Young Minds Matter Survey, children as young as four experience mental disorders, with rates rising as individuals approach adolescence (Lawrence et al., 2015).

Internalizing disorders, such as anxiety and depression, can have far reaching impacts on many areas of a child’s life, including school and family functioning, academic learning, self-esteem, and future quality of life (Kendall et al., 2004; Barrett et al., 2005; Lawrence et al., 2015). Externalizing disorders, such as ADHD and conduct disorder, affect relationships with family and peers, school success, and can contribute to problems in adulthood, such as increased risk of unemployment and criminal activity (Samek and Hicks, 2014). Internalizing and externalizing disorders also tend to have related or overlapping symptoms, and having one type can increase the risk of the other (Eisenberg et al., 2001; Samek and Hicks, 2014).

A meta-analysis of 41 studies from 27 countries investigated the global prevalence of mental health disorders in children and adolescents up to 18 years of age (Polanczyk et al., 2015). A worldwide pooled prevalence of 13.4% was reported with ADHD and any other disruptive disorder having a prevalence rate of 3.4% and 5.7%, respectively. Additionally, it was reported that anxiety and depressive disorders had a prevalence rate of 6.5% and 2.6%, respectively.

Regarding the prevalence of mental health disorders in younger children, a meta-analysis by Vasileva et al. (2021) investigated ten epidemiological studies from eight countries focusing on children between the age of 1 and 7 years. Vasileva et al. (2021) reported a pooled prevalence rate of mental health disorders of 20.1% with oppositional defiant disorder and ADHD being the most common disorders with a prevalence rate of 4.9% and 4.3%, respectively. Anxiety disorders and depressive disorders were reported to have a prevalence rate of 8.5% and 1.1%, respectively – with comorbidity having a prevalence of 6.4%.

It is estimated that 560,000 young people in Australia aged between 4 and 17 years have a mental health disorder (Lawrence et al., 2015). A national study found that 7.4% of young people aged 4–17 had a diagnosis of ADHD and 2.1% had a diagnosis of conduct disorder. Anxiety disorders and major depressive disorder were diagnosed in 6.9% and 2.8% of this demographic, respectively (Lawrence et al., 2015).

The prevalence and impact of mental disorders in young people have revealed a necessity for health services to target mental health symptoms early and screen for children who are potentially at-risk (Erskine et al., 2015; Patel et al., 2018). The literature suggests that emotion regulation difficulties may be implicated in the development of mental health problems (Cole et al., 1994; Loevaas et al., 2018) and as such, one way to intervene may be to specifically target and develop emotion regulation strategies in young children.

### Emotion Regulation and Coping

Emotion regulation refers to the processes that an individual uses to manage the way they experience and express their emotions (Eisenberg et al., 2010). Emotion regulation and coping are closely related concepts, both dealing with the processes which an individual uses to regulate themselves in response to their environment (Compas et al., 2014). In fact, it has been suggested that in relation to young children, emotion regulation and coping are terms that can be used interchangeably (Skinner and Zimmer-Gemback, 2007). Alternatively, other sources agree that coping is unique in that it involves regulatory processes in stressful situations, while emotion regulation applies to any situation (Compas et al., 2014). However, some of the literature also conceptualizes emotional regulation as being a type of coping (Compas et al., 2017). In this study, we will define emotional coping as the ability of an individual to use strategies to manage the strength and duration of the emotions that they experience, in particular uncomfortable emotions (Zeman et al., 2001).

Current theoretical models of emotion regulation and coping are mostly based on adults, and there is less literature on the way these models apply specifically to children in the early developmental years (Skinner and Zimmer-Gemback, 2007). Efforts have been made to study emotional coping from a more developmental perspective. Researchers have identified the different “families of coping” and explored the way in which these develop over infancy, childhood and adolescence (Skinner and Zimmer-Gemback, 2007). These “families of coping” refer to groups of strategies that children learn to use in order to regulate their emotions. Examples of these “families of coping” include support-seeking, problem solving, distraction and escape (Skinner and Zimmer-Gemback, 2007).

Gross (2015) offers a process model of emotion regulation suggesting that the strategies which individuals use to manage their emotions have different consequences for both the person and those around them, as well as varying levels of effectiveness in healthy emotional management (Gross, 2015). The model acknowledges that the way in which individuals cope with emotions influences wellbeing (Gross, 2015). The process model is similar to the literature on “families of coping,” as it describes groups of strategies that are used to regulate emotions and the way in which each family of strategies can be helpful or unhelpful to the individuals using them.

According to the process model, emotion regulation strategies can be grouped into five types of responses, categorized as situation selection, situation modification, attentional deployment, cognitive change and response modulation (Gross, 2015). Each of these groups of strategies falls along a different point of the emotion generation process.

Situation selection and situation modification occur near the beginning of the emotion generation process. Situation selection involves planning ahead to ensure that the individual finds themselves in situations that have the desired emotional consequences. Situation modification involves changing a situation to modify its emotional impact. While these strategies can be helpful, they can also be maladaptive, as they can contribute to avoidance behaviors and do not enable individuals to actively learn how to manage uncomfortable emotions (Gross, 2015).

Generally, the strategies used around the middle of the emotion generation process – attentional deployment and cognitive change – tend to be more adaptive, rather than those that aim to prevent emotions from occurring or suppress emotions that are already well developed (Gross, 2015). Attentional deployment is a healthy strategy that young children can use, it involves the child directing their attention elsewhere from the uncomfortable emotion they are currently experiencing (Skinner and Zimmer-Gemback, 2007; Gross, 2015). The use of attentional deployment strategies can be observed as early as infancy, and as such, are useful strategies to teach in early childhood (Skinner and Zimmer-Gemback, 2007). Specific strategies that fall under attentional deployment include techniques such as progressive muscle relaxation, exercise and deep breathing (Shafir et al., 2015). Cognitive change is another adaptive coping strategy which is generally effective in reducing the experience of uncomfortable emotions. This strategy involves changing the way a situation is appraised, and is used before the emotional response is well established (Gross, 2015). These groups of strategies work well together, for example, a child can initially use attentional deployment to reduce the intensity of the emotional experience, then later on use cognitive restructuring to further deal with the emotion.

Response modulation occurs at the end of the emotion generative process, and involves trying to influence an emotion after it has become well developed. Strategies in this group, such as substance use and expressive suppression, appear to be less effective for wellbeing (Gross, 2015). The process model suggests that it is important to ensure that children are given the opportunity to learn healthy rather than maladaptive ways of coping with their emotions, especially uncomfortable ones.

### Emotional Coping and Mental Health in Children

The past decades have seen a prolific increase in the literature on emotion regulation and its links to mental health (Hu et al., 2014; Gross, 2015). Research has explored the different strategies that individuals use to modify the duration, strength or quality of their emotional responses, finding that some strategies are more adaptive than others, and that it is possible for healthy strategies to be learnt (Gross, 2015). Evidence suggests that the way in which children cope with uncomfortable emotions is related to their risk of developing an internalizing or externalizing disorder (Zeman et al., 2002). Emotional coping can be observed as early as infancy, where babies are able to direct their focus to pleasant, rather than unpleasant stimuli (Skinner and Zimmer-Gemback, 2007). As children grow older, they continue to learn a wider repertoire of coping strategies, with development being particularly rapid during early childhood (Supplee et al., 2009; Chalmers et al., 2011).

Emotion regulation problems have been consistently linked to clinical disorders in both children and adults (Cole et al., 1994). A study found that a lack of regulation strategies was the strongest predictor for difficulties with mental health issues such as depression, anxiety and insomnia in comparison to a lack of goal directed behavior and trouble accepting emotional responses (Saxena et al., 2011). Teaching helpful emotion regulation strategies, such as cognitive appraisal, has been effective in the treatment of anxiety symptoms (Llewellyn et al., 2013). Effective management of emotions has been linked to greater psychological wellbeing, which suggests that effective coping strategies are needed to regulate emotions and prevent mental health disorders (Southam-Gerow and Kendall, 2002).

There is consistent evidence that both emotion regulation and coping problems are linked to internalizing and externalizing disorders in children and adolescents; this has been supported in a meta-analysis including 212 studies (Compas et al., 2017). Individual studies have explored these links from multiple aspects. A study with children aged 8–12 years who had either an anxiety disorder or no emotional difficulties found that the children with anxiety experienced emotions with intensity and reactivity, indicating a lack of regulation (Suveg and Zeman, 2004). Another study found that adolescents who used unhelpful emotion regulation strategies such as rumination and avoidance had increased depressive and anxiety symptoms, while those who used problem-solving and cognitive reevaluation had lower symptom levels (Schafer et al., 2017). Similarly, a cognitive behavioral intervention teaching children emotion regulation strategies found links between internalizing symptoms and decreased emotion regulation (Loevaas et al., 2018). Links have also been found between externalizing disorders and low emotion regulation (Supplee et al., 2009). Children with externalizing problems tend to have lower levels of emotion regulation (Eisenberg et al., 2001).

### Early Intervention Programs

The Australian Government has identified a critical gap in mental health intervention that spans the ages of birth to 12 years (National Mental Health Commission., 2014). It is particularly important to intervene early in life, as half of all mental illnesses begin before the age of 14 (World Health Organization., 2019). Given the potential of enhanced emotion regulation skills to target internalizing and externalizing problems, emotion-based interventions delivered to preschool children have great potential to address this gap. This may be especially important for young children who are already at risk due to early life experiences. Some children have a greater vulnerability to emotion regulation difficulties, due to internal and environmental risk factors (Supplee et al., 2009). Children who have experienced trauma may struggle more to cope with life events and the resulting emotions (Dozois et al., 2008; Dadomo et al., 2016). Experiences in early life, beginning *in utero*, have the capacity to affect a child’s mental and emotional wellbeing, and as such, it is especially important to provide early interventions for these children (National Mental Health Commission, 2014).

School-based preventative intervention programs are an optimal way to reach a large number of children. There are a few existing programs for preschool children that focus on building emotional skills, although not as many as there are for older primary and high school students (Stockings et al., 2016).

The *Emotions Course* (Izard et al., 2004) is a targeted program for children aged 3–4 years from low-income families. It aims to build emotional competence by teaching the identification and management of emotions, such as happiness, sadness, anger and fear. This is done using puppet demonstrations, discussions about emotions and emotion expression posters. Research conducted by Izard et al. (2004) employed a randomized-control trial with 116 children with a mean age of 3.8 years. Teachers of the intervention group delivered the program to their class over a 4-week period. The researchers found that children who participated in the program had reduced levels of negative emotional expression as compared to the control group (Izard et al., 2004). However, this study selectively targeted at-risk children, which could create stigma of labeling among the participants, as well as overlooking children who would also benefit from the program (Fisak et al., 2011).

In the *Early Heart-Smarts* (Bradley et al., 2012) intervention program, preschool children are taught how to understand and regulate emotions using strategies such as deep breathing exercises. The program is associated with significant increases in social and emotional health; however, a limitation was that it used observational methods of measurement only from the teacher, who was aware of the program’s aims.

The preschool *PATHS* program (Domitrovich et al., 2007) uses both parent and teacher reports to assess efficacy. The program targets externalizing behaviors, with sessions aimed to develop emotional awareness, self-control and problem solving. Domitrovich et al. (2007) employed a randomized clinical trial with 246 students with a mean age of 4.3 years. The program was universally delivered by teachers to their students over a 9-month period. Researchers found effects for emotional knowledge and social competence, but none for reducing aggressive behavior. However, this may be due to the analysis not controlling for nesting effects. The program focused more on social skills and self-control of behavior rather than strategies on how to regulate emotions. A limitation is that it did not target internalizing behaviors.

The *I Can Problem Solve* program (Anliak and Sahin, 2010), a behavior-change intervention for 5- to 6-year olds, targets externalizing behaviors and teaches social skills to decrease aggressiveness. The extensive 83 session program uses role-playing, puppets and other interactive activities to teach problem solving skills. A randomized-control trial of the program involving 83 students aged between 5 and 6 years required teachers to implement the entire program over a 4-month period (Anliak and Sahin, 2010). The study found significant improvements in inhibited and aggressive behaviors in the intervention group in comparison to the control group, as well as small effects on internalizing issues (Anliak and Sahin, 2010; Durlak et al., 2011).

The *Incredible Years* program (Webster-Stratton and Reid, 2003) aims to build children’s social and emotional skills, and decrease aggressive behaviors. Its primary focus is on teaching these strategies to parents to continue practicing at home. The program teaches children skills in emotional understanding, perspective taking, managing emotions, and communication skills. Although the focus was on externalizing behaviors, small improvements were found for internalizing issues (Durlak et al., 2011).

In summary, the literature indicates that promising interventions have begun to be developed for young children. However, few universal programs exist in the literature that teach young children emotion regulation strategies with the goal of preventing and reducing both internalizing and externalizing disorders. It is important to target both types of disorders, given the links between them and their impact on children. Universal programs have the advantage that they both decrease stigma and include all children, both low and high risk. Universal programs ensure that all children, including those at higher risk of poor mental health, are included in the intervention. Programs need to include developmentally appropriate strategies for coping with emotions.

### Aussie Optimism

The Aussie Optimism Program (AOP; Rooney et al., 2006; Pophillat et al., 2016; Roberts et al., 2018) is a suite of programs that aims to promote good mental health in children aged 5–13 years. Programs consist of 10 sessions to be universally delivered by teachers over a recommended 10 weeks with each session lasting an hour. Each program incorporates developmentally appropriate strategies to target internalizing and externalizing disorders in children. The programs are based on cognitive-behavioral principles and include emotion regulation strategies as a way of targeting poor mental health (Tennant et al., 2017). Coping strategies used across the programs include relaxation, facing fears, distraction and self-soothing (Pophillat et al., 2016). The programs use enactive programming, such as pleasant event scheduling to target depression, and gradual fear exposure to target anxiety (Morrison et al., 2013).

The first program in the existing suite is Feelings and Friends, of which there are two versions. The Feelings and Friends program for students in years 1 and 2 focuses on developing a basic emotional vocabulary. Students also learn about coping skills, asking for help and being friendly. The Feelings and Friends program for students in year 3 builds upon the first, but looks at emotions that are more complex and also includes content about social skills and problem solving. The second program, Positive Thinking Skills, is for students in year 4; it is based on cognitive behavioral strategies and has a heavy focus on developing a healthy thinking style. The program also looks at a range of coping strategies. The Social Life Skills program for students in years 5 and 6 focusses on developing social skills such as assertive communication, problem solving and negotiation. Students also learn the importance of having supportive social networks around them. The final program, Optimistic Thinking Skills, is for students in years 7 and 8, and is similar to the Positive Thinking Skills program. It focusses on challenging unhelpful thinking styles and developing optimistic thinking skills.

Each of these programs are structured and delivered in the same way. Trained teachers deliver the programs in a whole class context. The programs are designed so that students receive a one hour long lesson each week for 10 weeks. Each lesson is manualized and mapped to the health curriculum, and teachers are able to access coaching if they need support with implementation.

The programs aim to provide children with effective strategies to cope with stressful situations and manage their emotions. Efficacy testing has found that the programs contribute to reductions in depressive symptoms (Rooney et al., 2006, 2013), increased prosocial behaviors (Roberts et al., 2018), reductions in emotional difficulties (Rooney et al., 2013) and decreased suicidality (Roberts et al., 2018). Emotion regulation strategies have been included within programs; however, they have not been specifically measured in AOP efficacy studies to date.

The newest addition to the AOP suite is the I Spy Feelings Program, which has been especially developed for preschoolers. This program is suitable for children aged 4–6 years old and focuses especially on teaching children strategies for emotion regulation. Different emotions – such as anger, fear and sadness – are incorporated into the program’s modules. Children are taught how to cope with these emotions by using relevant regulation strategies, such as progressive muscle relaxation and deep breathing exercises.

### The Current Study

Healthy emotional coping is a key protective factor in the prevention of childhood mental illness. Research indicates that emotional coping strategies can be learnt and can have far reaching benefits to an individual’s mental health, especially if taught early in life (Compas et al., 2014). Research on the AOP has not yet focused mainly on the effects of emotion regulation strategies, nor has it investigated the efficacy of a program for preschoolers. The new Aussie Optimism: I Spy Feelings Program has been developed to address the current gaps in the literature, being a program especially for preschoolers that teaches them how to regulate their emotions.

The purpose of the current study was to assess the effects of the I Spy Feelings Program on emotional coping in children aged 5–6 years old. The study was part of a wider AOP research project; this branch of the project focused on emotional coping, operationalized as the ability of children to use strategies to manage their emotions. To test the efficacy of the program, a pre-test post-test cluster randomized controlled trial was used. We hypothesized that the I Spy Feelings AOP intervention would be associated with higher levels of emotional coping with sadness (H1a), anger (H1b) and worry (H1c) for children in the intervention group, compared to children in the control group.

## Materials and Methods

### Research Design

The current study was part of a larger Aussie Optimism pilot project testing the efficacy of the new preschool program. The research design employed was a pre-test post-test cluster randomized controlled trial, with a nested structure of students within schools. Four schools, each with one or two pre-primary classes, were randomly allocated to an intervention condition or a waitlist control condition, resulting in two schools in each condition. Pre- and post-testing were completed to determine if the program had an impact on the children’s emotional coping. The dependent variables in this study were the parent reported scores of children’s emotional coping with anger, sadness and worry. The independent variable was the I Spy Feelings Program, which children were either exposed to or not depending on whether their school was in the intervention or waitlist control condition.

### Participants

One hundred and sixty-nine pre-primary students aged 5–6 years from four Catholic primary schools in the Perth metropolitan area were invited to participate in this study. In Western Australia, pre-primary is the first year when students transition to being at school five full days a week. The school year before pre-primary is kindergarten, where children are only at school for half the time.

**FIGURE 1:**
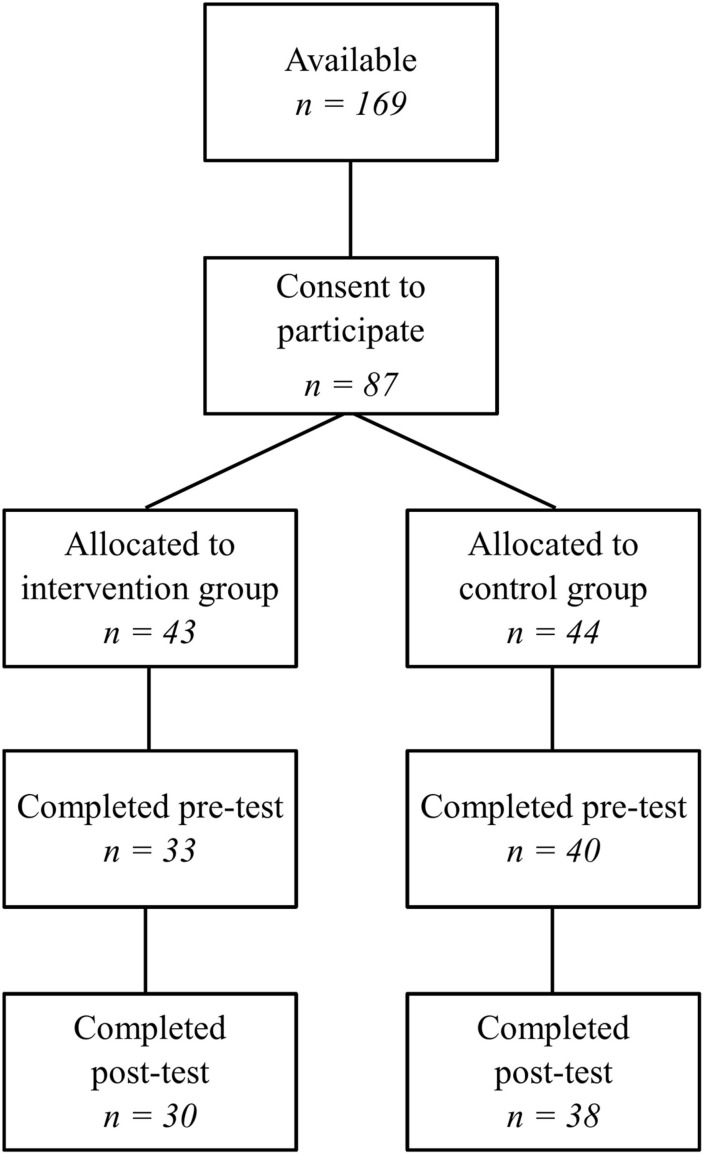
CONSORT diagram.

Expressions of interest were sought from all schools within Catholic Education Western Australia. After schools had indicated their interest, schools were matched into pairs based on size and socioeconomic status, and then four schools were randomly selected to be included in the project. One school from each matched pair was then randomly allocated to an intervention or a control group. Parental consent was obtained for 87 students; however, thirteen families did not return their questionnaires at pre-test, leaving a sample of 73 students. There were 33 children in the intervention group and 40 children in the control group, with a mean age of 5 years (Figure 1). At post-test the sample consisted of 68 children. Demographic information was collected about the students’ age, gender, ethnic origin, language, health and family financial situation (Table 1).

**TABLE 1:** Participant demographic information.

	Intervention	Control
*n*	33	40
Age	5.19	5.31
Gender	Male: 33.3%/ Female: 66.7%	Male: 65%/ Female: 35%
Ethnic origin		
ATSI	0%	0%
Australian/NZ (white)	36.40%	55%
North East Asian	6.10%	0%
Southern/Central Asia	3%	0%
Sub-Saharan Africa	3%	7.5%
North-West Europe	3%	0%
South-East Asia	15.20%	0%
Other/mixed race	33%	35%
United Kingdom	0%	2.5%
Language spoken at home		
English	69.7%	100%
English and one other	18.1%	
English and two other	3%	
Language other than English	9%	
Physical health problem in last 12 months	24.2%	20%
Physical or sensory disability	9.1%	7.5%
Mental health problem in last 12 months	3% unsure (97% = no)	2.5% unsure (95% = no; 2.5% = missing)
Financial situation at home		
Low income	9.1%	7.5%
Medium income	66.6%	57.5%
High income	21.2%	32.5%

The power analysis was conducted using G Power Version 3.1 (Faul et al., 2007) using an alpha level of 0.05. We estimated our sample size would be 158 students, to have an 80% chance of obtaining a small effect ($\eta_{\text{p}}^{2}$ = 0.01), and estimating the test-retest reliability of the scale to be similar to previous studies (Zeman et al., 2002, 2010). Small to moderate effects for short-term intervention programs can represent meaningful impacts (Ahlen et al., 2015); effects of these sizes have been found in previous AOPs (Morrison et al., 2013). Unfortunately, the desired sample size was not obtained. Of the students available, 51.3% agreed to participate, with 43.2% actually completing the pre-test.

### Measures

To assess emotional coping, we used the emotion regulation coping subscales from the Children’s Emotional Management Scale (CEMS; Zeman et al., 2002, 2010). These three subscales – one each for anger, sadness, and worry – are designed to be analyzed independently from the total CEMS (Zeman et al., 2001). The original versions of all three subscales were used.

The emotion regulation coping subscales measure the child’s ability to cope with anger, sadness and worry, using items such as “when my child is sad, he/she does something totally different until he/she calms down” and “my child tries to calmly deal with what is making him/her mad.” The items reflect adaptive strategies that children can use to cope with uncomfortable emotions. For each item, parents were asked to rate the frequency of their child’s behavior using a three-point Likert type scale (*1* = *hardly ever, 2* = *sometimes, 3* = *often)* on the anger, sadness, and worry scales.

The sadness scale contains five items, with a scoring range of 5–15. The anger scale includes four items, with a range of 4–12. Lastly, the worry scale contains three items, with a scoring range of 3–9. Higher scores on each of the emotion regulation coping scales indicate better emotional coping with that particular emotion (Zeman et al., 2002).

Cronbach’s alpha values for our sample indicated adequate reliability levels for the sadness (0.67) and worry (0.67) coping scales, and good reliability for the anger scale (0.75). Past studies that have used the parent-report version of the CEMS scales reported similar reliability levels (Sanders et al., 2015). For the number of items on these scales, these reliability coefficients are acceptable (Loewenthal, 2001). However, the sadness and worry scales were not as high in reliability as the anger scale.

Past research has found evidence of construct validity for the sadness coping subscale, as it correlates negatively with the Emotion Awareness Scale, as well as being inversely related to self-report measures of depression and anxiety (Zeman et al., 2001). The worry coping subscale has also demonstrated construct validity, being negatively correlated with the internalizing and externalizing subscales on the Child Behavior Checklist and positively correlated with a measure of emotion regulation (Zeman et al., 2010). The anger coping subscale is negatively correlated with externalizing symptoms (Zeman et al., 2002).

### Intervention

The I Spy Feelings Program was developed for 4- to 6-year-old children. It consists of 10 sessions (Table 2), each of which runs for approximately 40 minutes. Teachers are trained to deliver the program in class over a 10-week school term. The program is designed to be run as part of the regular health curriculum, with each activity specifically mapped to the Western Australian curriculum for Health and Physical Education (pre-primary).

**TABLE 2:** Outline of modules in the ‘I Spy Feelings’ Program.

Module	Name	Description
1	Feelings	For children to learn to communicate group rules, identify emotions and listen to others.
2	Feeling happy	Identifying body clues for feeling happy
3	Language for problem solving 1	Develop emotional vocabulary, cooperative play and following rules
4	Language for problem solving 2	Develop language to describe sequences and generating alternatives
5	Feeling sad 1	Developing language and body clues for feeling sad
6	Feeling sad 2	Develop coping strategies for feeling sad
7	Feeling scared 1	Develop language and body clues for feeling scared
8	Feeling scared 2	Develop coping strategies for feeling scared
9	Feeling angry 1	Develop language and body clues for feeling angry
10	Feeling angry 2	Develop coping strategies for feeling angry

Each of the ten sessions has a different focus but a similar session structure. For example, feeling sad is covered across two sessions, and this includes developing language to describe feeling sad, identifying body clues for feeling sad, and developing coping skills for feeling sad. This structure is similar for the other feelings explored in this program. Each session uses a variety of different activities to facilitate learning. For each session, parents also receive a printed handout explaining the content that their child has learnt in the lesson. The handout also contains activities that they can do at home with their child to support the learning in the classroom.

The intervention uses play-based learning to develop children’s social, emotional and cognitive abilities. The activities are age appropriate, such as stories and songs. There is a focus on developing language on emotions and problem solving. Children learn interpersonal skills as well as self-management skills. The vocabulary in the program is designed to be suitable for young children, such as talking about and noticing “body clues.” The program focuses on emotions that young children are likely to understand, namely, feeling happy, sad, scared, and angry. Children learn to recognize and cope with uncomfortable emotions.

### Procedure

Ethical approval for the larger Aussie Optimism I Spy Feelings study was granted by Curtin University’s Human Research Ethics Committee (HREC; approval no. HRE2018-0710) and the Catholic Education Office of Western Australia (CEWA). Informed consent was obtained from school principals, classroom teachers and parents; children signed an assent form after the research study had been explained to them by their parent or carer. Participating teachers in the intervention group attended a 4-hour training workshop conducted by the program developers. The workshop provided background information relating to mental health symptoms and statistics, social-emotional learning and the Aussie Optimism Program more broadly. Participants explored the materials of the I Spy Feelings program, sampled some activities and engaged in discussions around implementation and the rationale of the program. The workshop was designed to enable teachers to effectively run the program and support their students’ social-emotional development. The program developers offered ongoing support and coaching, however, this was not utilized by the participating schools.

Pre-test data was collected from parents. The parent version of the CEMS requires parents to select the response to each item that best describes their own child. No training is necessary to complete this questionnaire. After pre-testing had been completed, the teachers in the intervention group implemented the program in their classes, while the teachers in the control group conducted lessons as per the usual health curriculum. Due to delays in the roll out of the program, the pre-primary teachers implemented two modules of the program per week, completing it in 5 weeks. Post-testing commenced the following week – week 9 of the school term. Parents completed and returned their post-test questionnaires to teachers, who in turn sent them back to the researchers. Some parents completed their questionnaires within 2 weeks, while others took up to 5 weeks. Parents were not blind to group allocation. The parents in the intervention group were aware that their children were completing the program at the same time as the data collection; likewise, the control group parents were aware that their children had not completed the program yet but would participate in it after the study had concluded.

Teachers implementing the program completed records of fidelity. Each teacher filled in a logbook detailing the activities they used and whether any parts were omitted. The logbooks also included student attendance records for each lesson. All intervention teachers completed all ten modules of the program. Across all three teachers and ten modules, there were five times that an activity was missed due to time constraints. One teacher also did not complete the logbook for the activities in one module. Regardless of this, the results indicated that the large majority of activities were completed by teachers, representing a high level of fidelity. Teachers from two of the three classes reported student attendance. From these teacher reports, 44% of students attended all sessions, 20% attended nine, 85% attended eight, 24% attended seven, and 4% attended six. This means that although most students attended at least seven sessions, less than half of the students attended all of them.

Intervention schools initially planned to run a parent session as part of the program. However, this was unable to occur. Teachers asked parents to provide information on their interest and availability in order to organize the parent session; however, for both intervention schools, not enough parents responded to be able to organize the session. Feedback from parents indicated that they were not opposed to the idea but were not able to find the time.

In this study, the program took 5 weeks to complete, with teachers running two sessions each week as a part of regular school classes. The program is ideally run with one session per week; however, due to delays in ethics clearance, the program had to be condensed.

### Data Analysis

The three hypotheses were tested using generalized linear mixed models (GLMM), which were run using the SPSS Version 26 GENLINMIXED procedure. The use of GLMM was appropriate, as it takes into account violations of non-independence which result from using nested data. By specifying in the syntax that the data was multilevel, GLMM was able to accommodate intra-class dependencies in the dependent variables. In our analysis, we accounted for students being nested within schools. The GLMM analysis also adjusted for violations of normality and homogeneity of variance. Longitudinal data collection raises the issue of participant attrition. However, the GLMM maximum likelihood procedure decreases the need for missing data to be replaced as it uses all the data available at each data collection point, also decreasing sampling bias. Three GLMM analyses were run, using the sadness, anger and worry coping scores as the dependent variables. Each GLMM contained two nominal random effects (school, student), one nominal fixed effect (group) and one ordinal fixed effect (time).

## Results

Descriptive statistics were calculated for the sadness, anger, and worry scores for both groups at pre-test and post-test (Table 3).

**TABLE 3:** Descriptive statistics by emotion and group, over time.

Emotion	Time	Group	Mean	SE
Anger Coping Scale	Pre-test	Intervention	7.61	0.02
		Control	7.74	0.21
	Post-test	Intervention	7.68	0.19
		Control	7.36	0.24
Sadness Coping Scale	Pre-test	Intervention	9.33	0.36
		Control	9.46	0.13
	Post-test	Intervention	9.23	0.20
		Control	9.59	0.36
Worry Coping Scale	Pre-test	Intervention	6.84	0.13
		Control	7.02	0.10
	Post-test	Intervention	6.88	0.34
		Control	6.76	0.12

### Coping With Anger

**FIGURE 2:**
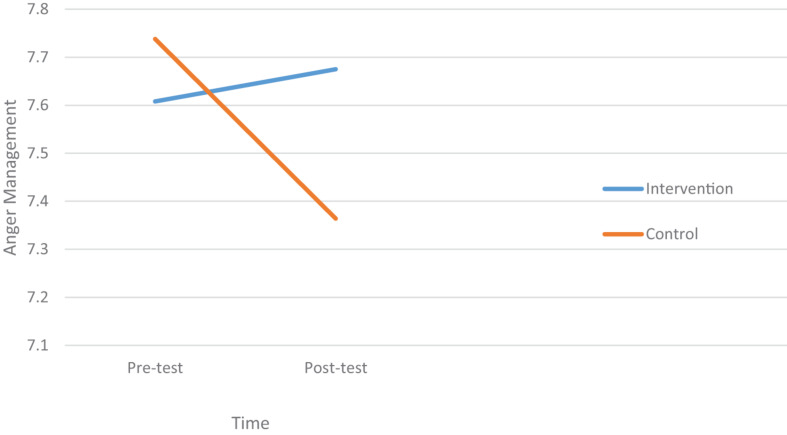
A plot of the simple main effect of time for the intervention and control groups for anger coping.

The Group × Time interaction was significant and small to moderate (*F*[1,137] = 6.39, *p* = 0.013, $\eta_{\text{p}}^{2}$ = 0.045) for Anger Coping. The source of the interaction was investigated by testing the simple main effect of time (Figure 2) within each of the two groups. There was a significant pre-post decrease in Anger Coping scores for the control group (*F*[1,137] = 230.75, *p* = 0.000); but no significant pre-post change for the intervention group (*F*[1,137] = 0.15, *p* = 0.699), as illustrated below (Table 4).

**TABLE 4:** GLMM results for anger, sadness, and worry coping.

	*Anger*		*Sadness*		*Worry*	
Source	*F[1,137]*	*Significance*	*F[1,136]*	*Significance*	*F[1,136]*	*Significance*
Corrected model	268.415	0.000	0.895	0.411	95.656	0.000
Time	3.10	0.081	0.003	0.953	1.12	0.291
Group	0.14	0.714	0.63	0.429	0.008	0.930
Group*Time	6.39	0.013	0.19	0.662	2.005	0.159

*Probability distribution: Normal.*

### Coping With Sadness

The Group × Time interaction was non-significant for Sadness Coping (*F*[1,136] = 0.19, *p* = 0.662, $\eta_{\text{p}}^{2}$ = 0.001). As the interaction was not significant, the main effects of Group and Time could be interpreted independently of each other (Table 4). The main effect of Time was non-significant (*F*[1,136] = 0.003, *p* = 0.953) as was the main effect of Group (*F*[1,136] = 0.63, *p* = 0.429).

### Coping With Worry

There was no significant Group × Time interaction for Worry Coping (*F*[1,136] = 2.005, *p* = 0.159, $\eta_{\text{p}}^{2}$ = 0.015). As this interaction effect was not significant, the main effects of Group and Time could be interpreted independently of one another (see Table 4). There was no difference between the two groups (*F*[1,136] = 0.008, *p* = 0.930), and no significant changes across time (*F*[1,136] = 1.12, *p* = 291).

## Discussion

The purpose of this study was to investigate if the I Spy Feelings Program would improve children’s emotional coping, specifically with the emotions of anger, sadness and worry. The results indicated that there was an intervention effect for coping with anger, but not for coping with sadness or worry.

The presence of an intervention effect for coping with anger supports hypothesis H1b, indicating that the program had a positive impact on this aspect of children’s emotional coping. Over the course of the study, students’ scores on emotional coping decreased in the control group, while no change was seen in the intervention group students, indicating a prevention effect. This suggests that the I Spy Feelings Program may contribute to the maintenance of children’s ability to cope with anger. This result provides support for the use of programs teaching emotion regulation strategies to help preschool aged children to manage their feelings. This result may be particularly relevant to the prevention of externalizing disorders, as the anger coping scale of the CEMS has previously demonstrated a relationship with externalizing symptoms (Zeman et al., 2002). Higher scores for coping with anger have been associated with lower levels of externalizing symptoms (Zeman et al., 2002). This result aligns with previous literature that suggests that children can learn effective coping strategies to manage uncomfortable emotions (Skinner and Zimmer-Gemback, 2007; Gross, 2015). In relation to the process model, although specific strategies were not measured in the anger coping scale, it appears that the items reflected strategies used toward the middle of the emotional cycle – such as attentional deployment – which tend to be more adaptive (Gross, 2015). This indicates that the program was effective in teaching children strategies to cope with anger. The prevention effect found is consistent with previous AOPs that found similar results with older students (Rooney et al., 2006). However, the current study also provides evidence that the strategies used in AOP are effective for children as young as 5 years of age.

Other studies of interventions with children of this age have found evidence for improvements in emotion regulation when using regulation strategies (Izard et al., 2004). The small to moderate effect size in our study is consistent with the effect sizes found in previous AOP’s as a public health intervention study (Morrison et al., 2013). Even small effect sizes can represent a considerable impact on the increasing burden of mental illness in children (Boiler et al., 2013). Given that this study was underpowered and yet still obtained a small to moderate effect, suggests that the program has practical utility in maintaining emotional coping with anger. Universal programs also tend to have smaller effect sizes than targeted programs, yet reach a larger number of students.

Another finding worth noting is that the students in the control group displayed a significant decrease in their ability to cope with anger over a period of only a few weeks. It appears concerning that emotional coping would decrease at this rate in such young children. On the other hand, pre-primary can be a challenging transition time as it is the first year of compulsory full-time schooling in Western Australia. As such, these results may indicate that pre-primary is the optimal time to implement such a program in order to prevent difficulties in emotional coping. This aligns with previous research which indicates that early childhood is a critical time for emotional development (Supplee et al., 2009).

The results for sadness and worry did not support H1a and H1c, as there were no significant intervention effects found at post-test. Children’s emotional coping for sadness and worry did not increase or decrease over time for either group in the study. There are a number of possible reasons that this did not occur, while there was an effect on anger. It may simply indicate that the program was not able to improve outcomes for these two emotions. Worry is also theoretically a harder emotion to identify than sadness and anger; it is not one of the six basic emotions (Sauter et al., 2010). Thus, it may be harder for both the child and parent to identify when the child is feeling worried and needs to cope with it. The worry scale also has fewer items than the other two scales, with other studies using the CEMS avoiding using it as it has less satisfactory reliability levels than the others (Sanders et al., 2015). The reliabilities for the sadness and worry scales in our sample was also not as high as for the anger scale. Although the reliabilities for the sadness and worry scales were adequate, they were lower than the ideal (Taber, 2016).

The lack of effects for sadness and worry coping can be used to inform the future development of the I Spy Feelings Program. More content on the emotions of sadness and worry could be incorporated into the modules. It is also possible that more than 5 weeks was necessary to see the effects of the program, and that a longer-term follow-up is needed to determine whether any effects were apparent later on. Usually, an Aussie Optimism program would be run over a period of 10 weeks, instead of five.

The differing results for the emotions of anger, sadness and worry could also be due to the nature of emotional problems across early and later childhood. Externalizing problems tend to occur earlier, and children who have externalizing problems in early childhood are at a higher risk of developing internalizing problems as they grow older (Wertz et al., 2015). With the anger coping scale corresponding more closely to externalizing issues, this could explain the decrease in coping for the control group students who did not learn the coping strategies in the program (Eisenberg et al., 2001; Zeman et al., 2002). The *I Can Problem Solve* program found effects for externalizing behaviors in a similar age group, suggesting that preschool is a good time for preventing externalizing disorders (Anliak and Sahin, 2010). In future studies of the I Spy Feelings Program, it would be beneficial to examine the effects on externalizing symptoms specifically. The sadness and worry scales correlate with internalizing symptoms (Zeman et al., 2001, 2010). The later onset of internalizing problems could explain why sadness and worry remained stable across both groups of students (Wertz et al., 2015).

Teachers delivered the sessions with a high level of fidelity, with all teachers delivering all modules of the program. The majority of activities in the modules were also delivered. There were only five instances of an activity being missed across all teachers and all modules. However, more than half of the students did not attend all the sessions, which could have influenced the lack of effects on sadness and worry. Future studies could find a way to increase student attendance in the program.

### Limitations and Future Directions

Our study had a number of limitations. Although the wider study included multiple informants, parents were the only informant in this current study, and may have been biased (Rapee and Jacobs, 2002). To mitigate this bias, future studies could use multiple informants to measure emotional coping. Parents may also have lacked information on emotional coping, due to the parent session not being run as planned. As mentioned earlier in this article, the teachers were not able to run the parent sessions due to a lack of availability among the parents in the intervention group. This may have impacted the parents’ understanding of appropriate emotionally regulated behavior, and their ability to help their children maintain the strategies they were taught in the program at school. The anger, sadness and worry scales used were not exhaustive in the number or type of healthy coping strategies included in the items. This means that children who used different strategies to the ones mentioned in the scales could have appeared lower in their coping ability than they actually were. For example, the sadness scale refers to strategies such as doing something different to calm down and managing crying (Zeman et al., 2001); however, some children may use other healthy strategies such as problem solving or trying to see the situation differently (Skinner and Zimmer-Gemback, 2007). Future studies could use a more comprehensive scale, or perhaps multiple different scales. As mentioned previously, the worry scale in the CEMS does not have high reliability.

Another limitation was that the demographics of the current sample were not representative of the wider population. None of the children were identified as coming from an Aboriginal or Torres Strait Islander background; however, in Western Australia, 6.5% of students come from an Indigenous Australian background (Commissioner for Children and Young People Western Australia., 2017). The sample was also not representative in terms of Culturally and Linguistically Diverse (CALD) students. Statistics indicate that 18.4% of children and adolescent were born overseas, and 10.6% speak a main language at home that is not English (Commissioner for Children and Young People Western Australia., 2017). Students from Indigenous and CALD backgrounds often face unique challenges that the rest of the student population does not, and as such, it is important to take these students into consideration when developing mental health programs. Future studies in Aussie Optimism should seek to include students from a more diverse range of cultural backgrounds.

Due to unforeseen circumstances, the implementation period for the current study was reduced from 10 to 5 weeks, resulting in two I Spy Feelings modules being implemented per week, rather than one. This had the limitation of providing less time for children to become familiar with the coping skills and emotion regulation strategies taught in the program, which could possibly have reduced their effectiveness. The sample size was also much lower than intended, due to the number of children participating being lower than expected. However, as a pilot study, research demands were hard to predict. Future research with pre-primary children and parents can be better informed on anticipated sample sizes. As the larger AOP project was a pilot study, future AOPs with this age group can mitigate the issues that emerged during this program.

To elaborate, in future, the research team will seek to recruit larger numbers of students, in anticipation of attrition during the study. The researchers will also broaden the range of schools approached, focusing on schools that have higher percentages of students from Aboriginal and Torres Strait Islander backgrounds, and CALD backgrounds. Schools in rural and remote Australia could also be approached, instead of only including schools from metropolitan areas in Perth.

The researchers will encourage teachers to deliver the program as intended, for example, with the parent session included and using the time frame of 10 weeks instead of condensing the program. This could be achieved in a number of ways – the researchers could go into schools and support teachers running the sessions, for example. The researchers could also run the teacher workshops earlier in the year to give teachers more time to prepare to run the program in their classrooms.

Our study found a significant intervention effect for coping with anger. This effect suggests that the I Spy Feelings Program may be able to prevent decreases in coping with anger among preschool children. This finding is important, as building healthy emotion regulation in children is a protective factor against internalizing and externalizing disorders, which have been reported in children as young as 5–6 years old (Lawrence et al., 2015; Compas et al., 2017). This study provides support for using programs to build emotional coping skills in preschool children. Developing emotional coping skills has been shown in the literature to decrease incidences of mental illnesses, thus it is important for these skills to be developed from a young age where these disorders are beginning to emerge (Compas et al., 2017). Teaching emotional coping strategies to 5- to 6-year-old children is beneficial to this age group, and future versions of the AOP should continue to use regulation strategies as a component of the program. This study did not find an intervention effect for sadness and worry coping, suggesting that the program could be modified to include more content on dealing with these emotions.

This pilot study tested the Aussie Optimism I Spy Feelings Program on a novel population of preschool children, while also being the first AOP study to focus on assessing emotional coping. As well as testing the outcomes, the research provided invaluable screening for children who had coping difficulties, enabling potential issues to be identified early in the child’s life. This study contributes to the research literature by building evidence for teaching emotional coping skills in preschool age groups. In conclusion, this study indicates that implementing programs that encourage the use of emotion regulation strategies has the potential to enhance children’s ability to cope with their emotions.

## Data Availability Statement

The raw data supporting the conclusions of this article will be made available by the authors, without undue reservation.

## Ethics Statement

The studies involving human participants were reviewed and approved by the Curtin University Human Research Ethics Committee and the Catholic Education Office of Western Australia. Written informed consent to participate in this study was provided by the children’s legal guardian/next of kin. Children provided written assent for their own participation in the study.

## Author Contributions

SO conceptualized the project, collected and analyzed the data, and was the main author of the manuscript. RR was the supervisor of the main author and assisted her throughout the research process, as well as contributing to the conceptualization of the project and the direction of the manuscript. NB coordinated the wider AOP project, including the data collection process. RK supervised the development of the research design and the analysis of the data. MM assisted in the analysis of the data and the preparation of the manuscript. AB collected the data and contributed to the writing of the manuscript. All authors contributed to the article and approved the submitted version.

## Conflict of Interest

The authors declare that the research was conducted in the absence of any commercial or financial relationships that could be construed as a potential conflict of interest.

## Publisher’s Note

All claims expressed in this article are solely those of the authors and do not necessarily represent those of their affiliated organizations, or those of the publisher, the editors and the reviewers. Any product that may be evaluated in this article, or claim that may be made by its manufacturer, is not guaranteed or endorsed by the publisher.
